# Urban green space visitation and mental health wellbeing during COVID-19 in Bangkok, Thailand

**DOI:** 10.3389/fpubh.2023.1292154

**Published:** 2024-01-16

**Authors:** Sigit D. Arifwidodo, Orana Chandrasiri

**Affiliations:** ^1^Department of Landscape Architecture, Faculty of Architecture, Kasetsart University, Chatuchak, Thailand; ^2^Activethai.org Research Center, Faculty of Architecture, Kasetsart University, Chatuchak, Thailand

**Keywords:** urban green space, landscape architecture, COVID-19, well-being, mental health, physical activity

## Abstract

Urban green spaces offer numerous benefits, and their role in supporting mental health, particularly during global crises such as the COVID-19 pandemic, is of growing interest to researchers and policymakers. This study explored the relationship between urban green space visitation and mental health well-being during the COVID-19 pandemic in Bangkok, Thailand. This cross-sectional study, conducted in Bangkok during the COVID-19 lockdown, used a telephone survey of 579 respondents. A logistic regression model was employed to examine the association between urban green space visitation and the WHO-5 mental health well-being score, considering various factors such as socioeconomic variables, healthy behaviors, and COVID-19-related experiences. The findings revealed a significant association between urban green space visitation during the lockdown and higher mental health well-being. Socioeconomic variables and healthy behaviors of respondents were also notably linked to higher WHO-5 mental health well-being scores. These findings collectively indicate that urban greenspace visitation serves as a crucial determinant of mental health and well-being, especially during the COVID-19 pandemic.

## 1 Introduction

The COVID-19 pandemic has had a profound impact on global health, causing not only physical illness, but also significant psychological distress. As countries worldwide have implemented lockdown measures to curb the spread of the virus, the mental health implications of these restrictions have become increasingly apparent. This has led to a growing body of research investigating the factors that may mitigate the adverse mental health and well-being effects of the pandemic. One factor that has received considerable attention is access to urban green spaces such as parks, gardens, and natural landscapes. They have long been recognized for their health benefits, such as providing opportunities for physical activity, social interaction, and contact with nature, all of which are known to enhance mental health and well-being (1–3). During the COVID-19 lockdown, when indoor recreational facilities were closed and opportunities for social interaction were limited, access to urban green spaces near homes became even more crucial (4). While lockdowns are necessary to curb the spread of the virus, they have significant mental health implications (5). Restriction of movement, combined with the closure of recreational facilities, has led to increased feelings of confinement, isolation, and stress (6). Previous studies conducted during the pandemic have highlighted the importance of green spaces in mental health. For instance, a study in the United Kingdom found that access to green space was associated with lower levels of psychological distress during the lockdown (4). Similarly, a study conducted in New York City found that respondents continued to use urban green spaces during the pandemic and considered them more important for mental and physical health than before the pandemic began (7).

Studies have shown a positive correlation between urban green spaces and mental well-being even before COVID-19. For instance, a study in Europe found that individuals living within 300 m of urban green spaces had a reduced risk of mood disorders and depression (8). Visiting urban green spaces is associated with mental health benefits such as recovery from mental fatigue and reduced stress (9). Living in greener environments has been consistently linked to improved mental health outcomes, including reduced stress, a lower risk of psychiatric disorders, and better cognitive development (10, 11). These studies provide consistent evidence that urban green spaces are positively associated with improved mental health and wellbeing. Extending this argument, it can be posited that during lockdowns, when movement is severely restricted, having access to urban green spaces becomes even more crucial for mental health well-being.

However, the relationship between access to green spaces and mental health well-being during the COVID-19 pandemic is not yet fully understood, and findings may vary depending on the specific context. In particular, there is a dearth of research on this relationship in Asian cities. Bangkok, a rapidly urbanizing city, presents a unique context in which to explore this issue. The dense population of the city, combined with the varying availability of urban green space across its districts, provides a valuable opportunity to investigate the potential mental health benefits of green space access during a period of unprecedented stress and disruption (12). On March 26, 2020, Bangkok implemented strong public health and social measures, including a full-scale city lockdown, curfews, and mandatory face masks, including the closure of places such as parks and recreational centers (13). The importance of urban green space visitation has increased with the closure of parks and public spaces. This paper argues that people who can still visit urban green spaces during the lockdown are positively associated with better mental health and well-being outcomes. In such a scenario, urban green spaces can act as buffers, providing residents with a connection to nature even if they cannot access larger public parks. The results of this study can provide policymakers and relevant stakeholders with a better understanding of the role of urban green spaces during and after the pandemic.

## 2 Materials and methods

This cross-sectional study was conducted during the lockdown period from March to May 2020. During this time, the Thai government implemented travel restrictions, curfews, and public-space closures. Consequently, face-to-face interviews were impossible.

A telephone survey was conducted to collect data. The respondents for the study were selected from a survey conducted in 2019 on park visits and physical activity in Bangkok.

In 2019, we conducted a survey of urban green spaces and physical activity in Bangkok. A quota sampling design was employed for the primary data collection by dividing the city into administrative districts. A list of registered households and their addresses was obtained from the Bangkok Metropolitan Administration (BMA) as the sampling frame. We randomly selected 12 respondents from 50 districts (600 respondents in total). We recruited a total of 10 surveyors to conduct face-to-face interviews with each respondent to ensure a 100% response rate. The respondents were given a token of appreciation for completing the interviews. The value of the souvenir was 100 Thai baht (THB), or approximately 30 US dollars (USD). During the survey, we collected a list of telephone numbers from respondents (with their consent).

This list was used to conduct the telephone survey. We believe that this method is more reliable than an online questionnaire using convenience sampling. Ten surveyors were recruited and trained to conduct telephone interviews in April, 2020. Participants were contacted by phone and informed of the study objectives and procedures. Once they agreed to participate, the surveyors asked them a set of structured questions divided into five main sections. These sections covered topics such as healthy behaviors during the lockdown, COVID-19 experiences, mental health and well-being, urban green spaces visitation, and socioeconomic characteristics. As a token of appreciation for their involvement, the participants received 100 THB (equivalent to approximately $30 USD) after completing the telephone interview. Cronbach’s alpha was used to ensure the reliability of the questionnaire, indicating a satisfactory level of internal consistency ranging from 0.79 to 0.84. After carefully cleaning the data, 579 responses were considered for the data analysis. The study followed the ethical principles outlined in the Declaration of Helsinki and was approved by the Institutional Review Board (or Ethics Committee) of the Institute for the Development of Human Research Protection (IHRP) in Thailand.

Previous studies have shown that healthy behaviors are important confounding variables when explaining mental health well-being during the COVID-19 pandemic. We collected four proxy variables related to healthy behaviors: physical activity level, presence of non-communicable diseases (NCDs), smoking, and alcohol consumption habits. Physical activity has been associated with mental health well-being during the COVID-19 pandemic. People who maintained an active lifestyle during the lockdown maintained stable levels of mental health. In this study, the level of physical activity was measured using the validated Global Physical Activity Questionnaire (GPAQ). This questionnaire assesses the amount of physical activity in three domains: work, transportation, and recreation. The GPAQ was administered by an interviewer and tailored to fit the Thai context based on previous studies conducted in Thailand (14, 15). For the analysis, we dichotomized the variables into sufficient and insufficient physical activity, following the global physical activity recommendation (16).

Non-communicable diseases (NCDs) have been found to be associated with mental health and well-being during COVID-19. Studies have indicated that individuals without NCDs may have better mental health and well-being than those with NCDs (17). This variable was measured by asking respondents whether they had NCDs with “yes” or “no” as possible responses. Smoking and alcohol consumption habits have been found to be associated with mental health and well-being during COVID-19 in previous studies. For example, a study in Australia found that negative changes in smoking and alcohol intake have been found to be associated with higher levels of depression, anxiety, and stress symptoms. These variables were measured by asking the respondents whether they regularly consumed alcohol and smoked during the lockdown, with “yes” or “no” as possible responses.

We also collected COVID-19-related experience variables, as suggested by previous studies, and found that COVID-19 exacerbates mental health and well-being. We collected data for two variables. The first variable was whether the respondents tested positive for COVID-19 or not. Previous studies have found that respondents who tested positive for COVID-19 were associated with mental health conditions, such as anxiety and depression (18–20). The second variable was whether respondents’ employment status was affected by COVID-19. Previous studies have found that having employment status affected by COVID-19, such as being fired from a job, having to change jobs, or work from home, is negatively associated with mental health well-being (21, 22).

The well-being variable was evaluated using the 5-item World Health Organization Well-Being Index (WHO-5). This index is a validated self-reported measure that gauges an individual’s subjective well-being over a period of 2 weeks (23). WHO-5 is widely used as a questionnaire to assess subjective psychological well-being and has been used in various studies to assess mental health in the context of environmental factors, making it an appropriate measure for this argument (24). The index comprises five positively worded items, each rated on a 5-point Likert scale ranging from 1 (none of the time) to 5 (all of the time), with a total raw score ranging from 5 to 25. However, it is customary to multiply the score by 4, resulting in a transformed score ranging from 0 to 100. A score below 50 indicates poor well-being and suggests the need for further investigation of the possible symptoms of depression (25, 26).

Urban green space visitation was assessed by inquiring whether respondents had visited urban green spaces within the last 2 weeks. In this study, urban green spaces were defined as spaces that remained accessible and open during the lockdown, allowing people to visit them, such as closed streets, neighborhood parks and gardens, community plazas, and other urban green areas that were not managed by the Bangkok Metropolitan Administration (BMA). During the lockdown, public parks and other green spaces managed by the government were closed. However, some parts of the Bangkok population still had access to urban green spaces, mostly because of their privileges of having good neighborhoods where privately owned public or community spaces were available (27). This variable was created following previous studies asking whether they had visited urban green spaces within the past 2 weeks during the lockdown, with “yes” or “no” as the possible responses. Previous studies in Bangkok have also used a similar proxy variable to measure urban green space visitation even before COVID-19 [for example, see (28, 29)]. The two-week timeframe was chosen to align with the time period used in the WHO-5 questionnaire. Moreover, we gathered information on socioeconomic factors such as income, education, sex, marital status, and the participants’ body mass index (BMI).

For the data analysis, we used descriptive statistics such as frequencies and means to summarize socioeconomic characteristics and other variables. To investigate the relationship between mental health well-being and independent variables, a multivariable logistic regression model was employed, presenting the results as odds ratios (ORs) with corresponding 95% confidence intervals (CIs). The model was adjusted for the potential confounding factors. All statistical analyses were performed using IBM SPSS Statistics version 24.

## 3 Results

Table 1 displays the summary statistics of the sample comprising 579 participants. The sample was predominantly composed of females (56.0%), unmarried individuals (59.6%), and those earning a monthly income ranging from 300 to 1,000 USD (46.1%). Only a small fraction of the respondents had postgraduate education, accounting for 5.8%. In terms of health-related behaviors, 11.4% of the participants acknowledged regular alcohol consumption, while 10% were regular smokers. The majority of the respondents (90%) reported the absence of non-communicable diseases. However, a notable proportion of participants (62.9%) indicated insufficient levels of physical activity, meaning that they engaged in 150 min or less of physical activity. Only 58.7% of respondents reported a WHO-5 score of more than 50 points. The data also showed that most of the respondents did not visit urban green spaces (60.3%).

**Table 1:** Respondents’ characteristics.

Category	Variable	Sample characteristics
Socioeconomic characteristics	**Monthly income**	
	Less than 5,000 THB (less than 160 USD)	30.1%
	5,000–10,000 THB (160–300 USD)	8.3%
	10,001–30,000 THB (300–1,000 USD)	46.1%
	30,001–50,000 THB (1000–1,600 USD)	9.5%
	More than 50,000 THB	6.0%
	**Education**	
	High school or less	47.0%
	High school to bachelor’s degree	47.2%
	More than bachelor’s degree	5.8%
	**Gender**	
	Male	44.0%
	Female	56.0%
	**Marital status**	
	Single	59.6%
	Living with partner	40.4%
COVID-19 related experience	**Ever tested positive for COVID-19**	
	Yes	28.0%
	No	72.0%
	**Employment status affected by COVID-19**	
	Yes	42.1%
	No	57.9%
Healthy behavior	**BMI**	
	25 or less	77.0%
	>25	23.0%
	No	88.6%
	**Regular smokers**	
	Yes	10.0%
	No	90.0%
	**Having non-communicable diseases (NCDs)**	
	Yes	10%
	No	90%
	**Physical activity level**	
	Physically active (more than 150 min/week)	37.1%
	Insufficient physical activity (150 min/week or less)	62.9%
Urban green spaces visitation during lockdown	**Visiting urban green spaces in the last 2 weeks**	
	Yes	39.7%
	No	60.3%
Mental health wellbeing	**WHO-5 score**	
	Less than 50	41.3%
	50 or more	58.7%

Table 2 shows the association between mental health well-being and access to green open spaces during the COVID-19 pandemic. The findings indicated that individuals who had access to urban green spaces during the lockdown period were more likely to have higher mental health well-being scores (OR = 2.001, *p* < 0.005). It was discovered that some socioeconomic variables were substantially correlated with the WHO-5 score. Higher education was associated with higher WHO-5 scores (high school to bachelor’s degree OR = 1.990, *p* < 0.005; higher than bachelor’s degree OR = 5.449, *p* < 0.005). Being female (OR = 0.520, *p* < 0.005) and having a BMI of >25 (OR = 0.520, *p* = 0.005) were associated with lower WHO-5 scores. COVID-19 related experiences were also found to be associated with WHO-5 scores. Respondents who had never tested positive for COVID-19 were more likely to have a higher score (OR = 1.937, *p* < 0.005). Respondents whose jobs were not affected by COVID-19 also had a higher WHO-5 score (OR = 1.201, *p* < 0.005). All healthy behavior variables, including not smoking or regular use of alcohol (OR = 1.439, *p* < 0.005), having sufficient physical activity (OR = 7.282, *p* < 0.005), and not having NCDs (OR = 2.108, *p* < 0.005), were positively associated with mental health well-being.

**Table 2:** Association between mental health wellbeing and access to green open spaces during COVID-19 pandemic.

Category	Variable	OR	95% CI
Socioeconomic characteristics	**Monthly income**		
	Less than 5,000 THB (less than 160 USD)	ref	
	5,000–10,000 THB (160–300 USD)	1.166	0.519–2.618
	10,001–30,000 THB (300–1,000 USD)	1.588	0.914–2.761
	30,001–50,000 THB (1,000–1,600 USD)	0.948	0.387–2.320
	More than 50,000 THB	0.806	0.260–2.504
	**Education**		
	High school or less	ref	
	High school to bachelor’s degree	1.990*	1.221–3.244
	More than bachelor’s degree	5.449*	1.520–19.527
	**Gender**		
	Male	ref	
	Female	0.520*	0.323–0.838
	**Marital status**		
	Single	ref	
	Living with partner	1.049	0.580–1.895
COVID-19 related experience	**Ever tested positive for COVID-19**		
	Yes	ref	
	No	1.937*	1.588–2.495
	**Employment status affected by COVID-19**		
	Yes	ref	
	No	1.201*	1.098–1.413
Healthy behavior	**BMI**		
	25 or less	ref	
	>25	0.469*	0.279–0.788
	**Regular alcohol consumption**		
	Yes	ref	
	No	1.439*	1.192–2.001
	**Regular smokers**		
	Yes	ref	
	No	3.364*	1.506–7.515
	**Having non-communicable diseases (NCDs)**		
	Yes	ref	
	No	2.108*	1.865–4.709
	**Physical activity level**		
	Insufficient physical activity (150 min/week or less)	ref	
	Physically active (more than 150 min/week)	7.282*	4.622–11.475
Urban green spaces visitation during lockdown	**Visiting urban green spaces in the last 2 weeks**		
	No	ref	
	Yes	2.001*	1.253–3.195

2 log likelihood = 566.708, chi-square = 218.248, *p < 0.005.

## 4 Discussion

This study examined the association between access to urban green spaces and mental health well-being during the COVID-19 pandemic. This study examined the association between access to urban green spaces and mental health and well-being during the COVID-19 pandemic. Two major findings were obtained in this study. First, the main finding of the study was that visiting urban green space during the lockdown period were significantly associated with mental health well-being. Even before the COVID-19 pandemic, visiting urban green spaces was positively linked to mental health outcomes (1, 30). During the COVID-19 lockdown, urban green spaces emerged as crucial sanctuaries for mental respite and well-being (31). A study in Italy found that more people wanted to visit urban green spaces during the lockdown period, and perceived urban green space as an important aspect that influences mental health and well-being (32, 33). In the UK and New Zealand, there is a significant increase in green space visits as lockdown restrictions eased, indicating people’s willingness to visit urban green spaces. Another study found that pedestrian activity increased in city parks, peri-urban forests, and protected areas in Kanazawa, Japan, during public park closures (34). Furthermore, studies have shown that visiting urban green spaces during the COVID-19 pandemic did not increase the risk of infection, although context-specific measures should be considered to ensure the safety of visitors (35, 36).

The fact that a part of Bangkok’s population still had the privilege to visit urban green spaces even during the government lockdown indicated that there was inequality in the ability to access urban green spaces. Inequalities in the use and experience of urban green spaces were found to exist even before the pandemic. Previous studies have examined this issue and found evidence of disparities based on socioeconomic status, race/ethnicity, and geographic location. Similar issues were also found in Bangkok, where parks were only accessible to groups of users (37). During the pandemic, inequalities in accessing urban green spaces were exacerbated (38). The closure of parks and green spaces during the pandemic has limited access to visitors and may affect vulnerable populations more than others. Low-income communities with less access to urban green spaces are often the same communities hardest hit by COVID-19 (17). On the other hand, people with better socioeconomic backgrounds would be less affected due to their neighborhood environment, which could provide alternatives to the closed parks and green spaces. Planned residential areas and condominiums provided green spaces and community areas that were still accessible to the residents even during the lockdown. This finding indicated that it is crucial to ensure that urban green spaces are accessible to all individuals, particularly during pandemics.

The second finding was that respondents’ socioeconomic and healthy behaviors variables were significantly associated with higher scores on the WHO-5 Mental Health Well-being Scale during the pandemic. Respondents with higher educational levels had higher mental health well-being scores. This finding suggests that individuals with higher education levels may have been more resilient and better equipped to cope with the challenges posed by the pandemic. Previous studies have also found a similar result, which may be because individuals with higher education levels may have had better access to and understanding of health information, leading to better mental health outcomes (39). Being female was associated with a lower mental health well-being score. Similar studies in China and the UK have found that women are more likely than men to report symptoms of depression and anxiety during the COVID-19 pandemic (39–41).

Healthy behaviors were also found to be important determinants of mental health well-being. We found that negative health behaviors were associated with lower WHO-5 mental health well-being scores. Individuals with obesity were linked to deterioration in their mental health during the lockdown, most likely due to changes in diet and eating behavior (42). We also found that the likelihood of a high mental health well-being score was reduced among those who regularly consumed alcohol and cigarettes during the pandemic. Prior studies have shown that lockdown increases alcohol intake and smoking at home, which negatively impacts mental health and well-being (43). Individuals with NCDs have been found to be particularly vulnerable to the impacts of external stressors, such as the COVID-19 pandemic (44). Although certain NCDs increased the severity of COVID-19 and mortality risk, previous studies have shown that containment measures, such as social distancing, quarantine, and lockdown, were the main factors that could potentially lead to increased mental health problems (45). We also found that sufficient physical activity is positively associated with well-being. Regular engagement in physical activities was found to be a protective factor against mental health challenges and enhance overall well-being in literature (46, 47). Studies have underlined the importance of being active at home, especially during the confinement period, because it would improve the mental health and well-being of an individual (48, 49).

This study has several limitations that warrant careful consideration when interpreting the findings. The cross-sectional design employed in this study precludes the establishment of a direct causal relationship between urban green space visits and mental health well-being. To establish a causal relationship, it is imperative to employ a more comprehensive research methodology, such as a longitudinal survey incorporating larger sample sizes and employing more rigorous probabilistic sampling techniques. Furthermore, the study employed a self-reported questionnaire administered through telephone surveys, which may have introduced potential biases. For instance, it is possible that participants may exhibit inaccuracies in their recollection or may tend to either overestimate or underestimate their visitation to urban green spaces, as well as their mental health well-being. The study also employed the WHO-5 as the exclusive measure of mental health well-being, potentially limiting its ability to assess an individual’s mental health outcomes comprehensively. Although the WHO-5 has been validated as a tool, it primarily captures the subjective perceptions of well-being. Divergent interpretations of a question by individuals can result in a range of responses. Furthermore, the study employed the concept of “Visiting urban green spaces during the period of lockdown” as a singular indicator for assessing the accessibility of urban green spaces. Various significant contributing factors, such as the proximity of these spaces, the frequency of visits, and the distribution of such spaces, were not measured in this study because of the difficulties in data collection during the lockdown. Additional research is necessary to employ more advanced and validated methodologies, incorporate a broader range of variables to assess mental health and well-being and evaluate the accessibility of urban green spaces beyond the lockdown period.

## 5 Conclusion

This study investigated the association between urban green spaces and mental health well-being during COVID-19 in Bangkok, Thailand. Our study revealed that visiting urban green spaces during the lockdown period was significantly associated with a higher WHO-5 mental health well-being score. Additionally, we also found consistent associations among socioeconomic variables and healthy behaviors of the respondents visiting urban green spaces, aligning with the existing literature, even before the pandemic. These findings collectively indicate that access to urban green spaces serves as a crucial determinant of mental health and well-being, especially during the COVID-19 pandemic. These findings reinforce the idea that equitable access to green spaces must be a key consideration in urban planning and public health strategies, as it has the potential to mitigate the adverse effects of stress, anxiety, and isolation that can accompany global crises, such as COVID-19. Prior research has highlighted increased mental health issues, especially among healthcare professionals and in vulnerable populations, as a result of the socio-economic consequences of the pandemic. As urban planners and policymakers grapple with the challenges posed by the pandemic, ensuring access to urban green spaces should be a priority, not just for recreational purposes but also as a vital component of public health.

While this study has limitations, such as the cross-sectional design and the use of self-reported measures, our results not only contribute to the understanding of the significance of urban green spaces for mental health but also highlight the persistent influence of healthy behaviors and socioeconomic factors in determining the mental health well-being outcomes. Future research should investigate the complex relationship between urban green spaces and mental health well-being using longitudinal data to track the long-term effects of frequent visits to urban green spaces on mental health well-being beyond the pandemic period. It’s also essential to investigate whether the association between urban green spaces and mental health varies after the pandemic compared to during it. Additionally, examining the effects of urban green spaces on diverse demographic segments, encompassing different ages, socio-economic statuses, and cultural backgrounds, is crucial for gaining a more detailed comprehension of these dynamics.

## Data availability statement

The raw data supporting the conclusions of this article will be made available by the authors, without undue reservation.

## Author contributions

SA: Conceptualization, Formal analysis, Methodology, Writing – original draft, Writing – review & editing. OC: Data curation, Funding acquisition, Investigation, Project administration, Resources, Writing – review & editing.
